# Expression levels of MCP-1, HGF, and IGF-1 in endometriotic patients compared with non-endometriotic controls

**DOI:** 10.1186/s12905-021-01560-6

**Published:** 2021-12-20

**Authors:** Sahel Heidari, Roya Kolahdouz-Mohammadi, Sepideh Khodaverdi, Nader Tajik, Ali-Akbar Delbandi

**Affiliations:** 1 Immunology Research Center, Institute of Immunology and Infectious Diseases, Iran University of Medical Sciences, Tehran, Iran; 2 Department of Immunology, School of Medicine, Iran University of Medical Sciences, Tehran, Iran; 3 Department of Nutrition, School of Public Health, Iran University of Medical Sciences, Tehran, Iran; 4 Endometriosis Research Center, Iran University of Medical Sciences, Tehran, Iran

**Keywords:** Endometriosis, MCP-1, HGF, IGF-1, PFMCs, Ectopic, Endometrial stromal cells

## Abstract

**Background:**

To study the concentrations of monocyte chemoattractant protein-1 (MCP-1), hepatocyte growth factor (HGF), and insulin-like growth factor-1 (IGF-1) in peritoneal fluid (PF) and serum, and to evaluate their expressions by PF and peripheral blood mononuclear cells (PFMCs and PBMCs, respectively), and ectopic and eutopic endometrial stromal cells of patients with endometriosis (EESCs and EuESCs, respectively) compared with controls.

**Methods:**

The concentrations of mentioned cytokines in serum and PF, as well as their expression in PBMCs, PFMCs, EuESCs and EESCs from endometriosis patients and controls were assessed.

**Results:**

The levels of MCP-1, HGF, and IGF-1 in serum and PF in women with endometriosis were significantly higher than the controls (*P* < 0.05–*P* < 0.001). Gene expression of *MCP-1* and *IGF-1* in the PFMCs, PBMCs and EESCs also showed an increased level compared to controls (*P* < 0.05*–P* < 0.01). The protein expression of MCP-1 and IGF-1 by PFMCs was statistically higher in endometriotic women (*P* < 0.05 and *P* < 0.01, respectively). The gene and protein expression of HGF in PFMCs and its gene expression by EESCs were significantly higher in endometriotic women compared to controls (*P* < 0.05–*P* < 0.01).

**Conclusions:**

The higher concentrations of mentioned cytokines in serum and PF and their higher expression by PFMCs and EESCs in endometriosis patients may contribute to the development of endometriosis.

**Supplementary Information:**

The online version contains supplementary material available at 10.1186/s12905-021-01560-6.

## Background

The presence of endometrial glands and stroma outside its normal site, the uterine cavity, is defined as endometriosis. Endometriosis is a common benign inflammatory disease that causes various symptoms such as chronic pelvic pain, dysmenorrhea, dyspareunia, and infertility [1]. The prevalence of endometriosis appears to have a range between 10 and 15% in the general population [2].

Recent studies have proven that endometriosis has a multifactorial etiology, with possible (epi)genetic, hormonal, and immunological factors as causes [3, 4]. Besides, based on a recent study, the loss of the integrity of the gastrointestinal barrier might contribute to the pathogenesis of endometriosis through an increase in lipopolysaccharides (LPS) concentration [5]. Many genes possibly implicated in different physiopathological molecular mechanisms of endometriosis like steroidogenesis, inflammation and immune response, tissue remodeling and neoangiogenesis, metabolism regulation and DNA reparation [3, 4].

Nevertheless, the most accepted theory for the etiology of endometriosis is Sampson's theory, which suggests endometriosis develops as a result of retrograde menstruation through the fallopian tubes [6]. However, retrograde menstruation occurs physiologically in almost 90% of healthy women, but less than one-fourth of them develop endometriosis. Studies have shown that immunological changes play a significant role in the pathogenesis of endometriosis, leading to incomplete elimination of endometrial cells and the increased ability of endometrial lesions to be created and implant in the peritoneal cavity [7]. However, the exact mechanism of endometriosis is unknown. Several changes in the number and function of various immunological components increase the volume of the peritoneal fluid (PF) in endometriotic patients. Evidence to date indicates mononuclear cells, especially macrophages, which constitute about 85% of the cells in PF, are more likely to cause inflammation and develop the disease rather than control it [8]. In addition to mononuclear cells, endometriosis may cause notable changes in the expression of different genes and proteins by eutopic endometrial stromal cells (EuESCs) and ectopic endometrial stromal cells (EESCs) [9]. Mononuclear cells, as well as EuESCs and EESCs, release cytokines and growth factors that can affect themselves and other cells, such as macrophages. These factors can promote proliferation, angiogenesis, and invasion of endometrial cells, the underlying fundamental mechanisms of the pathogenesis of endometriosis [10]. One of these factors is monocyte chemoattractant protein-1 (MCP-1). This chemokine activates and recruits macrophages and other mononuclear cells to secrete growth factors and cytokines. It also causes the proliferation and maintenance of endometrial cells in ectopic sites, and so, it may be involved in the pathogenesis of endometriosis [11].

Studies in women with endometriosis showed that hepatocyte growth factor (HGF) could also affect monocytes and macrophages and enhance inflammation. In addition to its growth-regulating properties, HGF has diverse impacts on epithelial and endothelial cells, such as proliferation, migration, extracellular matrix production, and tubulogenesis [12, 13]. Another mitogenic factor that is secreted by macrophages and other mononuclear cells is insulin-like growth factor-1 (IGF-1). Based on recent studies, EESCs can express the IGF-1 receptor immunohistochemically [14].

HGF and IGF-1 have several physiological and pathological effects that could contribute to the survival, proliferation, and invasion of endometrial stromal cells (ESCs) associated with endometriosis.

Increased concentrations of MCP-1, HGF, and IGF-1 have been reported in the PF and serum of endometriotic patients compared to controls in some studies [15–20]. In contrast, other studies failed to show significant differences in the concentrations of these factors between women with and without endometriosis [21–23].

The source of production of these factors is one of the controversial subjects in endometriosis; whether those originating from endometriotic lesions or are secreted by inflammatory mononuclear cells is unknown. These unclear data on MCP-1, HGF, and IGF-1 expression, hinder the understanding of the physiologic role of signaling in women with endometriosis, and no comprehensive study has examined all of the involved cells in endometriosis concurrently. In this study, we compared the concentrations of MCP-1, HGF, and IGF-1 in serum and PF of patients with and without endometriosis. Furthermore, we evaluated the expression of MCP-1, HGF, and IGF-1 by peritoneal fluid mononuclear cells (PFMCs), peripheral blood mononuclear cells (PBMCs), and ESCs in women with endometriosis compared to controls.

## Material and methods

### Participants

In the first step, 140 reproductive-aged women (24–40 years) took part in this study: 70 women with endometriosis (any stages of I–IV) and 70 patients with other benign gynecological disorders and without any evidence of endometriotic lesions in laparoscopy as a control. The diagnosis of endometriosis was made by laparoscopy and pathology reports, and the stage of disease was determined according to the revised American Fertility Society system [24].

Next, blood samples were taken from the participants, and their serum was stored at − 70 °C until protein expression. The demographic information of participants whose serum samples were collected is displayed in Table 1. In the second step, among mentioned participants, 36 women with stage III and IV endometriosis and 30 patients with benign gynecological disorders and no evidence of endometriotic lesions in laparoscopy were selected for collecting of PF, peripheral blood (to isolate mononuclear cells), and ectopic and eutopic endometrial tissues. The demographic information of participants whose PF samples were collected is displayed in Table 2. Besides, the demographic information of participants whose PFMCs, PBMCs, and ESCs were collected is displayed in Additional file 1: Table S1 and Table S2, respectively. Since some participants were virgins, the eutopic endometrial tissues were not collected from them.

**Table 1:** Demographic information of participants whose serum samples were collected

Characteristic	Endometriosis (n = 70)	Control (n = 70)	P_value_
Age (years)	32.7 ± 5.33 ^†^	32.1 ± 6.98	0.569
BMI (kg/m^2^)	24.3 ± 3.7	25.7 ± 5.45	0.069
Marital status, n (%)			
Single	18 (25.71)	17 (24.28)	0.845
Married	52 (74.28)	53 (75.71)	
Infertility among married, n (%)	18 (34.61)	12 (22.64)	0.174
Cycle phase, n (%)			
Secretory	29 (41.43)	32 (45.71)	0.609
Proliferative	41 (58.57)	38 (54.28)	
Stage, n (%)			
I & II	22 (31.42)		
III & IV	48 (68.57)		
Endometriosis type, n (%)			
Ovarian endometriosis (pure)	3 (4.29)		
Peritoneal endometriosis (pure)	8 (11.43)		
Tubo-ovarian & peritoneal endometriosis (including DIE)	59 (84.28)		

^†^Data are mean ± SD

Comparison was performed with Student’s t-test, or χ2- test, as appropriate

Abbreviations: BMI: Body mass index; DIE: Deep infiltrating endometriosis; n: Number

**Table 2:** Demographic information of participants whose peritoneal fluid samples were collected

Characteristic	Endometriosis (n = 36)	Control (n = 30)	P_value_
Age (years)	32.8 ± 5.64 ^†^	33.4 ± 5.69	0.656
BMI (kg/m^2^)	24.3 ± 3.46	25.2 ± 4.22	0.371
Marital status, n (%)			
Single	10 (27.78)	2 (6.67)	0.027
Married	26 (72.22)	28 (93.33)	
Infertility among married, n (%)	7 (26.92)	4 (14.28)	0.249
Cycle phase, n (%)			
Secretory	0 (0)	9 (30)	< 0.001
Proliferative	36 (100)	21 (70)	
Stage, n (%)			
I & II	0 (0)		
III & IV	36 (100)		
Endometriosis type, n (%)			
Tubo-ovarian & peritoneal endometriosis (including DIE)	36 (100)		

^†^Data are mean ± SD

Comparison was performed with Student’s t-test, or χ2- test, as appropriate

Abbreviations: BMI: Body mass index; DIE: Deep infiltrating endometriosis; n: Number

All subjects had a regular menstrual cycle, and patients with a history of malignancy, any acute or chronic diseases (especially autoimmune diseases), and using immunosuppressive drugs, hormones, or GnRH agonists for at least three months before sampling were excluded. We missed some samples due to gross bloody PF, culture contamination, not obtained the desired cells, or inconsistent pathology reports.

The study protocol was approved by the Ethics Committee of Medical Research of Iran University of Medical Sciences (Code: IR.IUMS.REC 1394.26098) and all methods were performed in accordance with the relevant guidelines and regulations. All subjects had written informed consent for participation in the study. The study was conducted regarding the privacy rights of all participants.

### Sample collection

Under sterile conditions, peritoneal ectopic endometrial patches were obtained through laparoscopic surgery, and eutopic endometrium samples were collected by uterine biopsy curettage. Endometrial tissues were placed in Dulbecco's modified Eagle's medium‐F12 (DMEM-F12) (Gibco, UK) culture medium containing 1% penicillin–streptomycin antibiotics (Gibco, Thermo Fisher Scientific, Waltham, MA, USA). Blood and PF samples were collected into EDTA-coated falcons. All samples were immediately transferred to a laboratory for analysis in the cold chain. To confirm endometriosis, parts of ectopic endometrial tissues were sent for pathologic evaluation.

### Mononuclear cell culture

PFMCs and PBMCs were isolated by density gradient centrifugation using Ficoll-Hypaque (Sigma-Aldrich, St. Louis, MO, USA). About 1 × 10^6^ cells/mL PFMCs or PBMCs were cultured in Roswell Park Memorial Institute medium (RPMI-1640) (Gibco, UK) supplemented with 10% fetal bovine serum (FBS) (Gibco, UK) and 1% penicillin–streptomycin antibiotic (Gibco, Thermo Fisher Scientific, Waltham, MA, USA).

### ESC culture

Endometrial tissues obtained from participants in both groups (endometriotic and non-endometriotic patients) were cut up into smaller pieces in dimension. Then tissue digestion was performed using collagenase-A (2 mg/mL) and DNase (300 µg/mL) (Roche, USA) for 120 min at 37 °C in 5% CO2 atmosphere with intermittent vortexing every 15 min. In an attempt to remove clots and undigested tissues, the suspension was filtered through a 100 µm mesh (BD Biosciences, San Jose, CA, USA). Then, cells were cultured in T25 culture flasks containing DMEM-F12 (Gibco, UK) supplemented with 10% FBS (Gibco, UK) plus 1% penicillin–streptomycin antibiotic (Gibco, Thermo Fisher Scientific, Waltham, MA, USA) for 6 h. Next, to remove non-adherent cells, they were washed twice with a warm medium, and adherent stromal cells were allowed to propagate. The cells in passage three were used for flow cytometry, immunofluorescence, RNA extraction, and enzyme-linked immunosorbent assay (ELISA). Also, 3 × 10^5^ cells/mL were cultured in a 24-well plate according to our previous study [25].

Flow cytometry and immunofluorescence were used to investigate the purity of the ESCs, and these cells were identified as vimentin^+^, nestin^+^ cytokeratin^−^, CD10^+^, CD44^+^, CD73^+^, CD105^+^, CD34^−^, and CD45^−^ (data not shown) [25].

### Total RNA extraction, complementary DNA (cDNA) synthesis, and quantitative real-time PCR reaction

RNA extraction was performed by QIAzol solution (Qiagen, Hilden, Germany) following the manufacturer's instructions. The Picodrop apparatus (Picopetol, Cambridge, UK) was applied to measure the concentration of total RNA at 260/280 nm. RNA integrity was confirmed by electrophoresis on 1% agarose gel. For cDNA synthesis, 1 μg RNA was used, and the cDNA was synthesized according to the Revert Aid First Strand cDNA Synthesis Kit (Thermo Fisher Scientific, Waltham, MA, USA) protocol.

Real-time PCR was performed using the Syber premix Extaq (Biofact, Daejeon, Korea) and Rotor-Gene Q (QIAGEN, USA). Each reaction was made up of 10 μL of Syber premix (Biofact, Daejeon, Korea), 1 μL of primer pairs, 1 μL of synthesized cDNA, and 8 µL of DNase-free water with a final volume of 20 μL. The concentration of the glyceraldehyde-3-phosphate dehydrogenase (GAPDH) primer was equal to the amount of the same primer for each reaction. Each PCR reaction was as follows: 95 °C for 15 min (holding step), 40 cycles of 95 °C for 20 s and 60 °C for 40 s (extension step), and finally, the melting step from 60 °C to 99 °C. To verify the real-time PCR results, melting curve analysis, and electrophoresis on 2% agarose gel were used. To increase the accuracy of real-time PCR, all analyses were performed in duplicate, and positive and negative controls were tested every time. The *GAPDH* gene was used as an internal control. The sequence of primers for *GAPDH*, *MCP-1*, *HGF*, and *IGF-1* genes are shown in Table 3.

**Table 3:** The MCP-1, HGF, IGF-1, and GAPDH primers sequences

Gene	Forward primer	Reverse primer	Amplicon size (bp)
MCP-1	*5′- GAAAGTCTCTGCCGCCCTT -3′*	*5′- TTGATTGCATCTGGCTGAGCG -3′*	84
HGF	*5′- GCAATTAAAACATGCGCTGACA -3′*	*5′- TCCCAACGCTGACATGGAAT -3′*	140
IGF-1	*5′- CTCTTCAGTTCGTGTGTGGAGAC -3′*	*5′- CAGCCTCCTTAGATCACAGCTC -3′*	134
GAPDH	*5′-GCACCGTCAAGGCTGAGAAC-3′*	*5′-TGGTGAAGACGCCAGTGGA-3′*	138

*Abbreviations*: bp: Base pair; GAPDH: Glyceraldehyde 3-phosphate dehydrogenase; HGF: Hepatocyte growth factor; IGF-1: Insulin-like growth factor-1; MCP-1: Monocyte chemoattractant protein-1

### ELISA procedure

The method used to measure the concentrations of MCP-1, HGF, and IGF-1 in PFMCs, PBMCs, and ESCs supernatant, as well as in serum and PF was sandwich ELISA (R&D Systems, Minneapolis, MN). The detection limit for MCP-1, HGF and IGF-1 were 15.6 pg/mL, 125 pg/mL, and 31.2 pg/mL, respectively. Each sample was analyzed in duplicate. The absorbance was measured at 570 nm using a microplate reader (Bio‐Rad, Hercules, CA, USA).

### Statistical analysis

All statistical analyses were conducted using GraphPad Prism software 8. The normality of distributions was evaluated by the Kolmogorov–Smirnov test. The independent t-test and Mann–Whitney U test were employed to compare two independent groups based on the normality distribution assumption and the chi-square test was used to assess categorical variables. For the comparison of three groups, Kruskal–Wallis test with Dunn post hoc analysis was used.

After normalization to GAPDH control, the quantitative analysis of mRNA expression was performed using the 2^−ΔΔCt^ method. *P* value < 0.05 was considered statistically significant.

## Results

In this study, serum concentrations of MCP-1, HGF, and IGF-1 were measured in 70 endometriotic and 70 non-endometriotic participants. Demographic data of the participants whose serum samples were collected are presented in Table 1. Based on our findings, no significant differences were observed between groups regarding age, body mass index (BMI), marital status, infertility, and menstrual phase. Besides, most patients who underwent surgery had stages III & IV of disease with deep infiltrative endometriosis, cul-de-sac obliteration, ovarian endometriosis, and dense adhesions. Superficial ovarian and peritoneal endometriosis or firm adhesions were observed in approximately 31 percent of patients who were in stages I & II. Regarding demographic information of participants whose PF, PFMC, PBMC, and ESCs were collected, no significant differences were observed between groups with concerning age, BMI, and infertility. All endometriotic patients were at the proliferative phase of the menstrual cycle and were at stage III & IV of endometriosis and had tubo-ovarian and peritoneal endometriosis (including deep infiltrating endometriosis (DIE)). The basal gene and protein expression of mentioned factors were measured in PFMCs (n = 10), PBMCs (n = 10), EESCs (n = 8), and EuESCs (n = 10) from endometriotic patients and PFMCs (n = 7), PBMCs (n = 10), and control endometrial stromal cells (CESCs) (n = 10) from non-endometriotic women. The relative expressions of *MCP-1*, *HGF*, and *IGF-1* were measured by quantitative real-time PCR, and the protein levels of these factors were evaluated by ELISA in PFMCs, PBMCs, and ESCs samples.

### Serum and PF concentrations of MCP-1 and its gene and protein expression by PFMCs, PBMCs, and ESCs

**Fig. 1:**
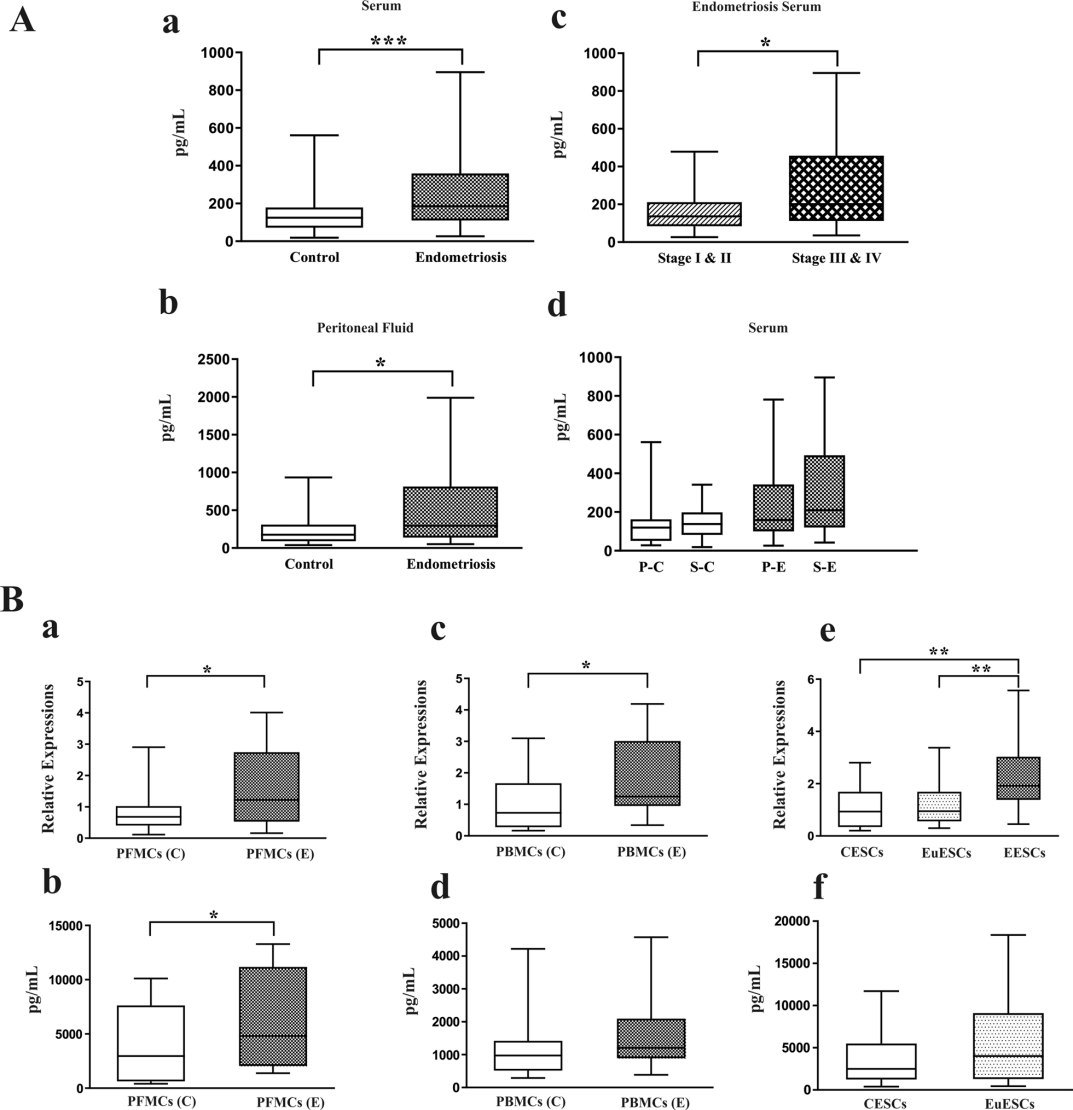
Serum and peritoneal fluid concentrations of MCP-1 and its gene and protein expression by PFMCs, PBMCs and ESCs. Serum concentrations of MCP-1 were measured in 70 endometriotic and 70 non-endometriotic participants. Peritoneal fluid concentrations of MCP-1 were measured in 36 endometriotic and 30 non-endometriotic participants. The basal gene and protein expression of MCP-1 were measured in PFMCs (n = 10), PBMCs (n = 10), EESCs (n = 8), and EuESCs (n = 10) from patients with endometriosis and PFMCs (n = 7), PBMCs (n = 10), and CESCs (n = 10) from non-endometriotic women. (**Aa**) serum concentration of MCP-1, (**Ab**) peritoneal concentration of MCP-1, (**Ac**) serum concentration of MCP-1 in different stages of endometriosis, (**Ad**) serum concentration of MCP-1 in different menstrual cycles, (**Ba**) *MCP-1* gene expression by PFMCs, (**Bb**) MCP-1 protein expression by PFMCs, (**Bc**) *MCP-1* gene expression by PBMCs, (**Bd**) MCP-1 protein expression by PBMCs, (**Be**) *MCP-1* gene expression by ESCs, (**Bf**) MCP-1 protein expression by ESCs. *P < 0.05, **P < 0.01, ***P < 0.001. A and B parts analyzed by parametric and non-parametric tests, respectively. ^†^P–C: Proliferative phase of the control group, S-C: Secretory phase of the control group, P–E: Proliferative phase of endometriosis patients, S-E: Secretory phase of endometriosis patients

The levels of MCP-1 in serum and PF were significantly higher in women with endometriosis than in controls (*P* < 0.001 and *P* < 0.05, respectively) (Figs. 1Aa and Ab). Also, the levels of MCP-1 in the serum were more remarkable in women with late-stages (III & IV) endometriosis than those with the early-stages (I and II) (*P* < 0.05) (Fig. 1Ac). Although, according to the menstrual phase, no significant difference in serum MCP-1 concentrations was noted in the endometriosis and control groups (Fig. 1Ad).

We further examined the gene and protein expressions of MCP-1 by PFMCs, PBMCs, and ESCs. There was a significantly higher MCP-1 expression at the level of mRNA and protein by PFMCs in women with endometriosis compared with non-endometriotic women (*P* < 0.05) (Figs. 1Ba and Bb). The increment in the mRNA expression of *MCP-1* in PBMCs in the patient group was significant, as well (*P* < 0.05) (Fig. 1Bc), while MCP-1 protein expression in PBMCs had no significant difference between women with and without endometriosis (Fig. 1Bd). EESCs showed increased gene expression of *MCP-1* compared to EuESCs and CESCs (*P* < 0.01) (Fig. 1Be), whereas MCP-1 protein expression was not different between EuESCs and CESCs (Fig. 1Bf).

### Serum and PF concentrations of HGF and its gene and protein expression by PFMCs, PBMCs, and ESCs

**Fig. 2:**
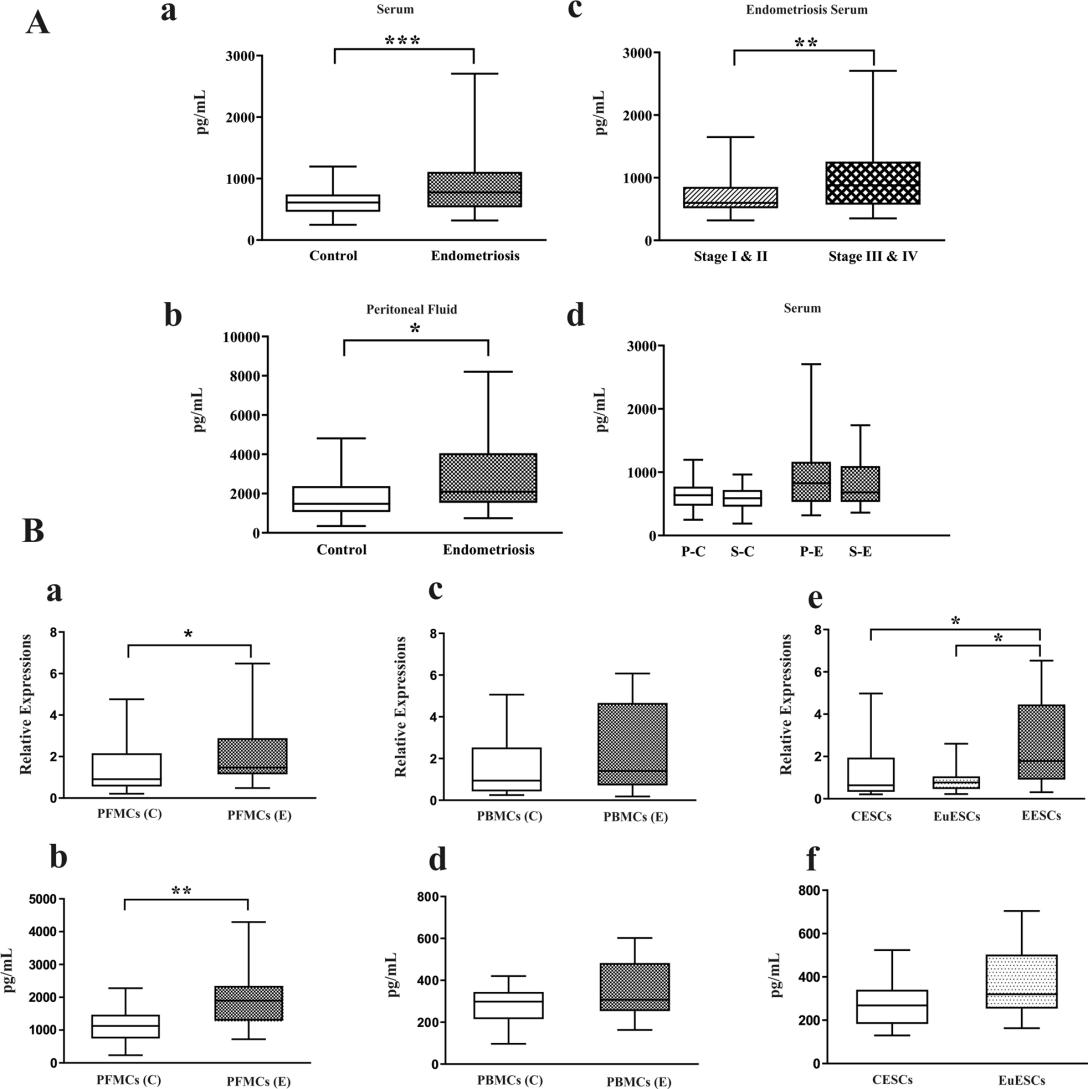
Serum and peritoneal fluid concentrations of HGF and its gene and protein expression by PFMCs, PBMCs and ESCs. Serum concentrations of HGF were measured in 70 endometriotic and 70 non-endometriotic participants. Peritoneal fluid concentrations of HGF were measured in 36 endometriotic and 30 non-endometriotic participants. The basal gene and protein expression of HGF were measured in PFMCs (n = 10), PBMCs (n = 10), EESCs (n = 8), and EuESCs (n = 10) from patients with endometriosis and PFMCs (n = 7), PBMCs (n = 10), and CESCs (n = 10) from non-endometriotic women. (**Aa**) serum concentration of HGF, (**Ab**) peritoneal concentration of HGF, (**Ac**) serum concentration of HGF in different stages of endometriosis, (**Ad**) serum concentration of HGF in different menstrual cycles, (**Ba**) *HGF* gene expression by PFMCs, (**Bb**) HGF protein expression by PFMCs, (**Bc**) *HGF* gene expression by PBMCs, (**Bd**) HGF protein expression by PBMCs, (Be) *HGF* gene expression by ESCs, (**Bf**) HGF protein expression by ESCs. *P < 0.05, **P < 0.01, ***P < 0.001. **A** and **B** parts analyzed by parametric and non-parametric tests, respectively. ^†^P–C: Proliferative phase of the control group, S-C: Secretory phase of the control group, P-E: Proliferative phase of endometriosis patients, S–E: Secretory phase of endometriosis patients

The results showed that the level of HGF was significantly higher in the serum and PF in women with endometriosis than in controls (*P* < 0.001 and *P* < 0.05, respectively) (Figs. 2Aa and Ab). Additionally, there was a significantly higher difference in the serum concentration of HGF in women at the stages III-IV of endometriosis compared to the patients with the stages I-II of the disease (*P* < 0.01) (Fig. 2Ac). No difference between the follicular or luteal phases in terms of serum levels of HGF was detected in the patient and control groups (Fig. 2Ad).

The expression of HGF gene and protein by PFMCs was significantly higher in the patient's group compared to the controls (*P* < 0.05 and *P* < 0.01, respectively) (Figs. 2Ba and Bb). However, there was no notable difference in their expression by PBMCs in both groups (Figs. 2Bc and Bd). *HGF* gene expression was significantly higher in EESCs compared with the EuESCs and CESCs (*P* < 0.05) (Fig. 2Be). However, we did not find any difference in the HGF protein expression between EuESCs and CESCs (Fig. 2Bf).

### Serum and PF concentrations of IGF-1 and its gene and protein expression by PFMCs, PBMCs, and ESCs

**Fig. 3:**
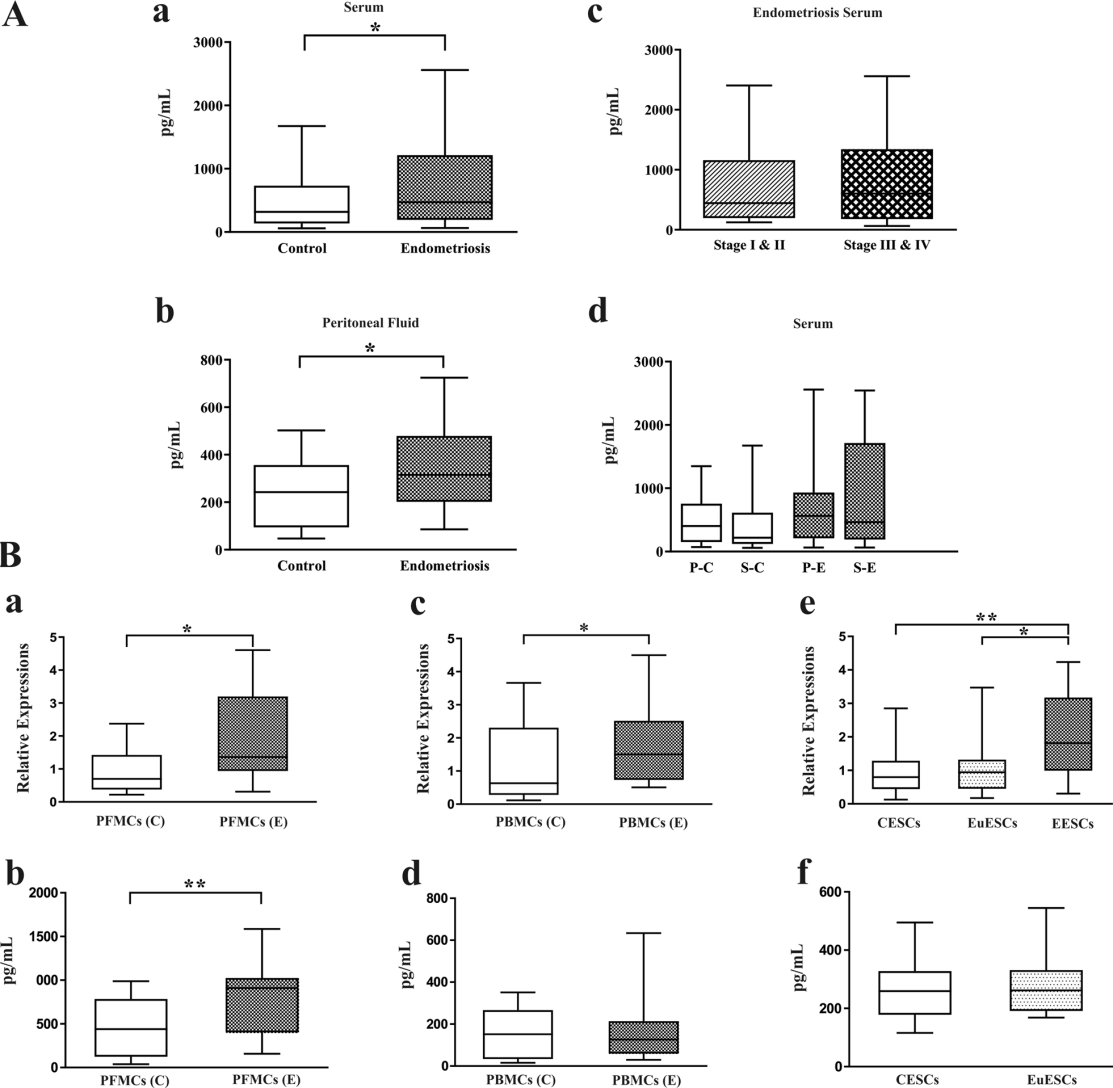
Serum and peritoneal fluid concentrations of IGF-1 and its gene and protein expression by PFMCs, PBMCs and ESCs. Serum concentrations of IGF-1 were measured in 70 endometriotic and 70 non-endometriotic participants. Peritoneal fluid concentrations of IGF-1 were measured in 36 endometriotic and 30 non-endometriotic participants. The basal gene and protein expression of IGF-1 were measured in PFMCs (n = 10), PBMCs (n = 10), EESCs (n = 8), and EuESCs (n = 10) from patients with endometriosis and PFMCs (n = 7), PBMCs (n = 10), and CESCs (n = 10) from non-endometriotic women. (**Aa**) serum concentration of IGF-1, (**Ab**) peritoneal concentration of IGF-1, (**Ac**) serum concentration of IGF-1 in different stages of endometriosis, (**Ad**) serum concentration of IGF-1 in different menstrual cycles, (**Ba**) *IGF-1* gene expression by PFMCs, (**Bb**) IGF-1 protein expression by PFMCs, (**Bc**) *IGF-1* gene expression by PBMCs, (**Bd**) IGF-1 protein expression by PBMCs, (**Be**) *IGF-1* gene expression by ESCs, (**Bf**) IGF-1 protein expression by ESCs. *P < 0.05, **P < 0.01. A and B parts analyzed by parametric and non-parametric tests, respectively. ^†^P–C: Proliferative phase of the control group, S-C: Secretory phase of the control group, P–E: Proliferative phase of endometriosis patients, S–E: Secretory phase of endometriosis patients

Results obtained showed that the IGF-1 level in serum and PF in women with endometriosis was higher than in women without endometriosis (*P* < 0.05) (Figs. 3Aa and Ab). An increase in the serum levels of IGF-1 in women with stages III-IV endometriosis was observed compared to women with stages I-II endometriosis, but it was not statistically significant (Fig. 3Ac). Also, no obvious difference was detected among the IGF-1 serum concentrations in the different phases of the menstrual cycle of women with endometriosis and controls (Fig. 3Ad).

It is demonstrated that a substantial amount of IGF-1 was also produced by PFMCs at the level of mRNA and protein in women with endometriosis (*P* < 0.05 and *P* < 0.01, respectively) (Figs. 3Ba and Bb). *IGF-1* gene expression by PBMCs in endometriosis patients was significantly higher than in women without endometriosis (*P* < 0.05) (Fig. 3Bc). However, there was no difference in the secretion of IGF-1 by PBMCs in the two groups (Fig. 3Bd). The *IGF-1* gene expression by EuESCs and CESCs was significantly lower than EESCs (*P* < 0.05 and *P* < 0.01, respectively) (Fig. 3Be). But, the levels of IGF-1 protein expression did not significantly differ between the EuESCs and CESCs (Fig. 3Bf).

## Discussion

Endometriosis is a complex disease with systemic and topical immune system defects. Sampson’s theory of retrograde menstruation is well accepted theory for intraperitoneal and ovarian endometriosis [6]. However, it cannot account for the less common locations of endometriosis, like remote areas. Remote endometriosis is likely caused by bone marrow-derived stem cells (BMDSCs) [26] and epigenetics play a major role in modulating the key factors involved in BMDSCs recruitment and differentiation [27]. Besides, microbiome may be involved in the pathogenesis of endometriosis as increased Proteobacteria, Bacteroidetes, and Negativicutes levels were shown in a recent systematic review [28]. On the other hand, macrophages are involved in tissue remodeling during the development of endometriosis. Lagana et al. for the first time showed a progressive decrease in M1 macrophages from stage I to stage IV; in contrast, M2 macrophages showed a progressive increase from stage I to stage IV. These findings may contribute to pro-inflammatory microenvironment typical of the early stages of the disease and pro-fibrotic nature of the advanced stages of endometriosis [29]. Besides, invariant natural killer T cells (iNKT) which are capable of secreting Th1 and Th2 cytokine patterns, may be involved in the pathogenesis of endometriosis [30]. Many studies have noted that changes in chemokines and immune receptors result in the development and progression of this disease in features such as increased proliferation, angiogenesis, invasion, and decreased apoptosis of ectopic cells [7]. Some essential chemokines are MCP-1, HGF, and IGF-1, which play a crucial role in the proliferation and invasion of ESCs.

In the current study, the serum and peritoneal levels of MCP-1 were higher in women with endometriosis than in control subjects, and the severity of the disease, unlike the menstrual cycle, was directly related to the concentration of this factor. These results are consistent with the findings of some previous studies [15, 16, 31, 32], although there are studies that have shown no difference between the two groups of patients and controls [33–35]. However, Margari et al. reported remarkably lower concentrations of MCP-1 in the PF of patients with endometriosis [36]. The findings of this study showed that MCP-1 gene and protein expression in PFMCs increased more markedly in patients with endometriosis compared to controls. Also, *MCP-1* gene expression was substantially higher in EESCs and PBMCs of women with endometriosis compared to EuESCs, CESCs, and PBMCs of control groups. These results are in line with other studies which showed that endometrial epithelial cells of women with endometriosis express high levels of MCP-1 [37, 38], and EESCs expressed more MCP-1 than EuESCs and CESCs [39].

MCP-1 is one of the critical factors that have the potent ability not only in the infiltration of monocytes into the inflammatory site and their differentiation, but also in stimulating macrophages to secrete chemokines and cytokines. So, increased macrophage activation and recruitment into the peritoneal cavity of patients with endometriosis are considered to progress chronic inflammation and inflammatory cytokines production [40]. Increased MCP-1 secretion has been demonstrated by PF macrophages in patients with endometriosis compared with controls that affect monocytes and macrophages via autocrine manner [41]. In addition to macrophages, MCP-1 is secreted by endometrial, peritoneal mesothelium, mononuclear cells, endothelial cells, and fibroblasts. It can lead to the infiltration of monocytes, macrophages, eosinophils, natural killer (NK), and T cells and may contribute to the shift to TH2 response [40, 42]. MCP-1 directly contributes to the proliferation and survival of cancer cells [43], and the similarity of endometriosis with malignant diseases has been noted in features such as increased proliferation, angiogenesis, invasion, and decreased apoptosis of ectopic cells [44]. So, we suggest that MCP-1 may be involved in proliferation, survival, and invasion of EESCs, and it can increase the expression of CCR2 and MCP-1 and result in a defective cycle and more activation and recruitment of peritoneal macrophages in PF via autocrine and paracrine mechanisms.

HGF is a multi-functional and essential growth factor that, by binding to its receptor (c-Met), results in various effects, several of which are potentially related to growth and proliferation, invasion, and metastasis in cancer cells [18, 45]. We observed higher concentrations of HGF in serum and PF of patients with endometriosis compared to controls, and its concentrations were higher at the late stages of endometriosis. No difference was noted regarding HGF serum levels with the menstrual cycle. Many studies have indicated increased HGF levels in PF and serum of endometriotic patients and evidenced that this elevation was significant in the late stages of the disease [15, 18, 20, 46]. Besides, no significant difference was observed in HGF serum concentrations in endometriotic women in the follicular or luteal phases in those studies. In contrast, in one study, no significant difference was observed between the two groups of patients and controls [21]. In one study, it has been illustrated that the expression of c-Met is related to the different stages of endometriosis [47].

Our current study showed that PFMCs in women with endometriosis could produce HGF considerably more than controls, and EESCs expressed substantially high levels of HGF than EuESCs and CESCs.

So far, few studies have been conducted regarding the production of HGF by PFMCs and PBMCs in patients with endometriosis, and some available studies have demonstrated the release of HGF by ESCs of endometriosis patients. Sugawara et al. showed increased HGF secretion by EuESCs in women with endometriosis compared with controls [48]. However, in this research, EESCs were not studied. Nasu et al. investigated HGF secretion in endometrial cell culture media and showed that HGF secretion was probably via the protein kinase C pathway [49]. According to other studies, it was revealed that HGF expression and c-Met in eutopic endometrium in patients with endometriosis increased compared to controls [50]. In line with our results, other studies have shown a significant increase in the gene expression of *HGF* in EESCs compared to EuESCs and CESCs [25, 51].

HGF plays a significant physiological role in the proliferation of various cell types. Other studies have also proven that the proliferation of ESCs and macrophages in patients with endometriosis in response to HGF in the culture medium had a more significant increase than the control group. The enhanced capacity of ESCs and macrophages proliferation may reflect more co-expression between HGF and its receptor in the cells of endometriosis patients [52]. The relationship between high expression of fibroblast activation protein and HGF with angiogenesis and metastasis in gastric cancer has been reported [53]. HGF also plays a crucial role in the development and progression of many tumor cells. Noguchi and colleagues showed that binding HGF to its receptor increased angiogenesis in tumor cells, which ultimately leads to increased cell proliferation, migration, and invasion of gastric cancer cells [54].

Regarding the malignancy-like nature of endometriosis and the high concentration of HGF in the PF of patients with endometriosis, it is reasonable to speculate that HGF can play a role in the pathogenesis and progression of the endometriosis. Consequently, increasing HGF secretion by PFMCs and EESCs leads to increased inflammation in the region, proliferation, and invasion of ESCs.

IGF-1 is another important factor involved in the growth and proliferation of ESCs, which, along with increased estrogen receptor B and aromatase expression, lead to the progression of endometriosis [55]. This study also showed increased IGF-1 concentration in serum and PF of patients with endometriosis, but no correlation was found regarding the stage of disease or the phase of the menstrual cycle with serum levels of IGF-1. Previous studies consistent with ours showed increased concentrations of IGF-1 in serum and PF in patients with endometriosis compared with controls [17, 56]. Although, some studies reported no significant difference in IGF-1 levels in the serum of women with endometriosis compared to control subjects [23, 57]. According to our literature review, no studies have ever been done on the production of IGF-1 by PFMCs and PBMCs in patients with endometriosis. One study revealed an increased IGF-1 gene and protein expression in EESCs of patients with endometriosis [25]. Rutanen et al. showed that ESCs produced IGF-1 and IGF-1 binding protein (IGFBP), and that was associated with levels of sex hormones and the menstrual cycle [58].

Milingos and colleagues examined *IGF-I* isoforms in ESCs and showed that the CESCs expressed lower *IGF-1* compared to EuESCs and EESCs [59].

We demonstrated that the probable source of IGF-1 is PFMCs and EESCs in women with endometriosis resulted in increased levels of this factor in PF and the uncontrolled growth of EESCs.

It has been reported that the peritoneal IGF-1 level is about 60% of its serum level. Studies have shown that IGF-1 affects on ESCs in the culture, and it is an influential factor in the growth and proliferation of ectopic endometrium. Therefore, IGF-1 might be one of the most critical factors in women with endometriosis [60].

So increased IGF-1 levels in the peritoneum of these patients appear to be involved in the pathogenesis of endometriosis, and in particular, in infertility.

Our study revealed that the primary sources of MCP-1, HGF, and IGF-1 are probably PFMCs and EESCs, which lead to a regional inflammatory environment and, by creating a defective cycle, contribute to the progression of the disease. As based on the recent findings it has been shown that, after retrograde menstruation, refluxed endometrial cells located outside the uterus stimulate the infiltration of immune cells into lesions, which secrete inflammatory mediators (like, pro-inflammatory cytokines, and chemokines) and these factors activate the nuclear factor kappa B (NF-κB) pathway, and NF-κB further increases transcription of multiple genes encoding pro-inflammatory cytokines, chemokines and angiogenic factors like MCP-1, HGF, and IGF-1, finally resulting in an inflammatory peritoneal microenvironment. So this cocktail of secretions in PF leads to intensification of inflammation [61, 62]. In this study, despite the increased expression of *MCP-1* and *IGF-1* in PBMCs of patients with endometriosis compared to controls, we observed no significant difference in their protein levels, and this can be due to post-transcriptional changes in mRNA and RNA degradation for various reasons [52].

This contradiction at the level of transcription and protein production requires future studies.

One limitation we faced within this study was the inability to evaluate MCP-1, HGF, and IGF-1 proteins in EESCs, because of the small number of EESCs that was due to the specific nature of EESCs and their difficult growth condition, so it should be examined in other studies. Furthermore, in future studies, other factors associated with growth, invasion, and angiogenesis in endometriosis and different materials with the suppressive effect on those, could be evaluated.

## Conclusion

We conclude that PFMCs, as well as ESCs in women with endometriosis, can express a large amount of MCP-1, HGF, and IGF-1 factors, indicating the important role of these factors in the pathology of endometriosis and their possible involvement in the development of endometrial lesions in the ectopic site.

## Supplementary Information

**Additional file 1**. **Table S1.** Demographic information of participants whose PBMCs and PFMCs were collected. **Table S2.** Demographic information of participants whose ESCs were collected.

## Data Availability

The datasets used and/or analyzed during the current study are available from the corresponding author on reasonable request.

## Abbreviations

BMDSCs: Bone marrow-derived stem cells
BMI: Body mass index
cDNA: Complementary DNA
CESCs: Control endometrial stromal cells
DIE: Deep infiltrating endometriosis
DMEM-F12: Dulbecco's modified Eagle's medium‐F12
EESCs: Ectopic endometrial stromal cells
ELISA: Enzyme-linked immunosorbent assay
ESCs: Endometrial stromal cells
EuESCs: Eutopic endometrial stromal cells
FBS: Fetal bovine serum
GAPDH: Glyceraldehyde-3-phosphate dehydrogenase
HGF: Hepatocyte growth factor
IGFBP: IGF-1 binding protein
IGF-1: Insulin-like growth factor-1
iNKT: Invariant natural killer T cells
LPS: Lipopolysaccharides
MCP-1: Monocyte chemoattractant protein-1
NF-kb: Nuclear factor kappa B
NK: Natural killer
PBMCs: Peripheral blood mononuclear cells
PF: Peritoneal fluid
PFMCs: Peritoneal fluid mononuclear cells
RPMI: Roswell Park Memorial Institute medium
