# Supplementary material for: Expression levels of MCP-1, HGF, and IGF-1 in endometriotic patients compared with non-endometriotic controls
Source: BMC Womens Health. 2021 Dec 20;21:422. doi: 10.1186/s12905-021-01560-6 (PMC8686524; doi:10.1186/s12905-021-01560-6)
Supplement: Supplementary file 1 — Additional file 1. Table S1. Demographic information of participants whose PBMCs and PFMCs were collected. Table S2. Demographic information of participants whose ESCs were collected. [file 12905_2021_1560_MOESM1_ESM.docx]

**Additional Table 1** Demographic information of participants whose PBMCs and PFMCs were collected

| P_value_ | Control (n=10) | Endometriosis (n=10) | Characteristic |
| --- | --- | --- | --- |
| 0.419 | 31.7±7.72 | 34.3±6.27 ^†^ | **Age (years)** |
| 0.844 | 25.2±5.47 | 24.8±3.52 | **BMI (kg/m^2^)** |
|  |  |  | **Marital status, n (%)** |
| 0.606 | 3 (30) | 2 (20) | *Single* |
|  | 7 (70) | 8 (80) | *Married* |
| 0.310 | 1 (14.28) | 3 (37.5) | **Infertility among married, n (%)** |
|  |  |  | **Cycle phase, n (%)** |
|  | 0 (0) | 0 (0) | *Secretory* |
|  | 10 (100) | 10 (100) | *Proliferative* |
|  |  |  | **Stage, n (%)** |
|  |  | 0 (0) | *I & II* |
|  |  | 10 (100) | *III & IV* |
|  |  |  | **Endometriosis type, n (%)** |
|  |  | 10 (100) | *Tubo-ovarian & peritoneal endometriosis (including DIE)* |

^†^ Data are mean ± SD

Comparison was performed with Student’s t-test, or χ2- test, as appropriate.

Abbreviations: BMI: Body mass index; DIE: Deep infiltrating endometriosis; n: Number, PBMCs: Peripheral blood mononuclear cells; PFMCs: Peritoneal fluid mononuclear cells.

**Additional Table 2** Demographic information of participants whose ESCs were collected

| P _value_ | Control (n=10) | Endometriosis (n=10) | Characteristic |
| --- | --- | --- | --- |
| 0.570 | 29.2±7.97 | 31±5.77 ^†^ | **Age (years)** |
| 0.987 | 23.8±4.65 | 23.8±3.83 | **BMI (kg/m^2^)** |
|  |  |  | **Marital status, n (%)** |
|  | 3 (30) | 3 (30) | *Single* |
|  | 7 (70) | 7 (70) | *Married* |
| 0.299 | 0 (0) | 1 (14.28) | **Infertility among married, n (%)** |
|  |  |  | **Cycle phase, n (%)** |
|  | 0 (0) | 0 (0) | *Secretory* |
|  | 10 (100) | 10 (100) | *Proliferative* |
|  |  |  | **Stage, n (%)** |
|  |  | 0 (0) | *I & II* |
|  |  | 10 (100) | *III & IV* |
|  |  |  | **Endometriosis type, n (%)** |
|  |  | 10 (100) | *Tubo-ovarian & peritoneal endometriosis (including DIE)* |

^†^ Data are mean ± SD

Comparison was performed with Student’s t-test, or χ2- test, as appropriate.

Abbreviations: BMI: Body mass index; DIE: Deep infiltrating endometriosis; ESCs: Endometrial stromal cells; n, Number.
